# C-Reactive Protein as an Early Predictor of Efficacy in Advanced Non-Small-Cell Lung Cancer Patients: A Tumor Dynamics-Biomarker Modeling Framework

**DOI:** 10.3390/cancers15225429

**Published:** 2023-11-15

**Authors:** Yomna M. Nassar, Francis Williams Ojara, Alejandro Pérez-Pitarch, Kimberly Geiger, Wilhelm Huisinga, Niklas Hartung, Robin Michelet, Stefan Holdenrieder, Markus Joerger, Charlotte Kloft

**Affiliations:** 1 Department of Clinical Pharmacy and Biochemistry, Institute of Pharmacy, Freie Universität Berlin, 12169 Berlin, Germany; yomna.nassar@fu-berlin.de (Y.M.N.);; 2 Graduate Research Training Program PharMetrX, Berlin/Potsdam, Germany; 3 Department of Pharmacology, Faculty of Medicine, Gulu University, Gulu P.O. Box 166, Uganda; 4 Translational Medicine & Clinical Pharmacology, Boehringer Ingelheim Pharma GmbH & Co. KG, 55216 Ingelheim am Rhein, Germany; 5 Institute of Laboratory Medicine, German Heart Centre Munich of the Free State of Bavaria, Technical University Munich, 80636 Munich, Germany; 6 Institute of Mathematics, University of Potsdam, 14476 Potsdam, Germany; huisinga@uni-potsdam.de (W.H.); niklas.hartung@uni-potsdam.de (N.H.); 7 Department of Medical Oncology and Hematology, Cantonal Hospital, CH-9007 St. Gallen, Switzerland

**Keywords:** non-small-cell lung cancer, C-reactive protein, biomarkers, prognosis, nonlinear mixed-effects modeling, time-to-event analysis, overall survival, progression-free survival

## Abstract

**Simple Summary:**

In oncology, the identification of early predictors of response/survival is of particular interest. C-reactive protein (CRP) concentrations have been associated with advanced non-small-cell lung cancer and poor prognosis. We characterized the association between anticancer drug exposure, tumor size as a marker of tumor dynamics, and CRP as a marker of inflammation and derived different predictors. CRP at the beginning of treatment cycle 3 (day 42) was identified as the strongest predictor of both progression-free survival and overall survival, and the inflammatory status, monitored by CRP concentration, emerged as a promising prognostic marker. The high significance of longitudinal CRP concentrations compared to baseline concentrations provided a true reflection of the patient status. This framework could be applied to other treatment modalities such as immunotherapies or targeted therapies, allowing the identification of patients at risk of early progression and/or short survival to spare them unnecessary toxicities and offer alternative treatment decisions.

**Abstract:**

In oncology, longitudinal biomarkers reflecting the patient’s status and disease evolution can offer reliable predictions of the patient’s response to treatment and prognosis. By leveraging clinical data in patients with advanced non-small-cell lung cancer receiving first-line chemotherapy, we aimed to develop a framework combining anticancer drug exposure, tumor dynamics (RECIST criteria), and C-reactive protein (CRP) concentrations, using nonlinear mixed-effects models, to evaluate and quantify by means of parametric time-to-event models the significance of early longitudinal predictors of progression-free survival (PFS) and overall survival (OS). Tumor dynamics was characterized by a tumor size (TS) model accounting for anticancer drug exposure and development of drug resistance. CRP concentrations over time were characterized by a turnover model. An x-fold change in TS from baseline linearly affected CRP production. CRP concentration at treatment cycle 3 (day 42) and the difference between CRP concentration at treatment cycles 3 and 2 were the strongest predictors of PFS and OS. Measuring longitudinal CRP allows for the monitoring of inflammatory levels and, along with its reduction across treatment cycles, presents a promising prognostic marker. This framework could be applied to other treatment modalities such as immunotherapies or targeted therapies allowing the timely identification of patients at risk of early progression and/or short survival to spare them unnecessary toxicities and provide alternative treatment decisions.

## 1. Introduction

Lung cancer is the second most common cancer type and the leading cause of cancer-related death [1]. Amongst all lung cancer cases, non-small-cell lung cancer (NSCLC) accounts for approximately 85% of these cases. At the time of diagnosis, the majority of NSCLC patients are at an advanced stage when the tumor has already metastasized, which consequently leads to patients having a very poor prognosis and an expected 5-year survival of only <7% [2]. This high disease burden and poor prognosis calls for markers that can timely predict patients with poor response to treatment as well as identify patients at risk of poor prognosis, for better treatment decisions. 

Past research has focused on the identification of several (bio)markers to predict treatment response and patients at increased risk of recurrence and/or poor prognosis in NSCLC [3,4]. Serum/blood biomarkers offering a prognostic value in NSCLC are appealing given that their collection through blood sampling is minimally invasive and their measurements are cost-effective. However, the focus has usually been on baseline measurements when assessing the prognostic value of serum/blood biomarkers rather than their longitudinal measurements [5,6,7,8,9]. The latter would have the added advantage of not only looking into the kinetics but also taking into consideration the influence of treatment and disease evolution over time on the relevant markers. Amongst the potential markers that have been previously investigated is C-reactive protein (CRP), a systemic inflammatory marker and a metric of inflammatory response. CRP is produced by the hepatocytes in response to the release of the inflammatory cytokines, e.g., interleukin 6 (IL-6) [10], and high concentration (>10 μg/mL) has been associated with advanced cancer stages, metastasis, and poor prognosis [11] in different cancer types including NSCLC [6,7,12,13,14,15]. Although the causal relationship between inflammation and advanced tumor disease is still unclear [11,16], the association of an inflammatory state with malignancy has been described [11,17,18]. 

Pharmacometric modeling and simulation (M&S) represents a multidisciplinary science integrating biology and mathematical and statistical methods to characterize, understand, and predict the behavior of a biological system (here NSCLC patients receiving drug treatment) through the development of a simplified representation of the complex drug-biological system interaction (i.e., the pharmacometric model) [19]. M&S can not only impact drug development through guidance on dosing selection but also clinical practice by predicting clinical response, optimizing the benefit–risk assessment, and guiding dosing strategies and therapy decisions through model-informed precision dosing [20]. The integration of pharmacokinetic (e.g., drug exposure) and/or pharmacodynamic models (e.g., tumor dynamics, biomarkers) with survival analyses, within a modeling framework, offers a powerful approach to monitoring the efficacy of treatment and predicting clinical outcomes, accounting for and quantifying the heterogeneity and variability within the patient population that M&S offers [21].

Our objective was to (a) develop a framework to understand and quantify the relationship between anticancer drug exposure, tumor dynamics, and CRP to (b) identify early longitudinal prognostic predictors of the commonly used clinical efficacy endpoints, progression-free survival (PFS), and overall survival (OS), in advanced NSCLC patients treated with first-line platinum-based chemotherapy. We also focused on longitudinal CRP metrics in combination with patient- and treatment-specific information to explore whether monitoring inflammation, through CRP, would reflect disease outcomes. These objectives were achieved by leveraging clinical data from patients with advanced NSCLC using pharmacometrics M&S principles, specifically nonlinear mixed-effects and parametric time-to-event models to allow for further exploration of the performance of the identified predictors under different conditions.

## 2. Materials and Methods

### 2.1. Clinical Data

Data from an open-label, randomized, two-arm, phase III, multicenter study—the CEPAC-TDM study, were used to establish the quantitative relationship between anticancer drug exposure, tumor dynamics, and CRP concentration and to identify significant predictors of efficacy endpoints thereafter [22]. A total of 365 patients with newly diagnosed advanced NSCLC were treated with paclitaxel (3-h intravenous infusion) in combination with either carboplatin (target AUC = 6 mg·min/mL) or cisplatin (80 mg/m^2^) on day one of each of the three-week-long (21-day) cycle, for up to six cycles. In the standard body surface area (BSA)-guided dosing arm, 182 patients received standard paclitaxel doses of 200 mg/m^2^, while in the pharmacokinetic (PK)-guided dosing arm, 183 patients received individualized paclitaxel doses according to a developed algorithm based on paclitaxel exposure and grade of neutropenia from the previous cycle [23]. PK sampling was performed in only the PK-guided dosing arm patients, 24 h (16–30 h) after the start of paclitaxel infusion.

Routine blood sampling was performed at baseline, on days 1 and 15 of each treatment cycle, and at the end-of-treatment (EOT) visit (27–35 days after the last treatment dose) for assessment of safety.

Tumor size (TS), defined as the sum of the longest diameters of a maximum of five target lesions, was evaluated according to the Response Evaluation Criteria in Solid Tumours (RECIST) criteria (version 1.1) [24], and was measured at baseline and subsequently every six weeks, i.e., before the start of treatment cycle 3 and cycle 5, at the EOT, and then every eight weeks during follow-up, until progression was observed. 

Serum CRP concentrations over time were available from the biomarker substudy of the CEPAC-TDM study including 258 patients. CRP concentration was measured on day 1 of treatment cycles 1 (baseline), 2, and 3; day 2 of treatment cycles 1 and 2; and at the EOT visit. CRP quantification was performed at the Institute of Laboratory Medicine, Munich Biomarker Research Center, German Heart Center Munich, Technical University Munich/Germany using a validated commercial assay-based latex-enhanced turbidimetry on the Cobas C501 analyzer (Roche Diagnostics, Mannheim, Germany). 

Patients were followed up every three months for survival. According to protocol, patients who dropped out during the study were still followed up for information on progression and survival.

The study was approved by the respective institutional review boards (Ostschweiz, St. Gallen/Switzerland; Eberhard-Karls-Universität, Tübingen/Germany) and conducted in accordance with the ICH Harmonised Tripartite Guidelines for Good Clinical Practice 1996, Directive 91/507/EEC [25], Declaration of Helsinki, Directive 2001/20/EC [26], and local legislation (EUDRACT 2010-023688-16). All patients provided written informed consent before study initiation. Further details on the study, inclusion and exclusion criteria, and the dosing algorithm, have been previously published [22,23].

### 2.2. Modeling Framework

**Figure 1:**
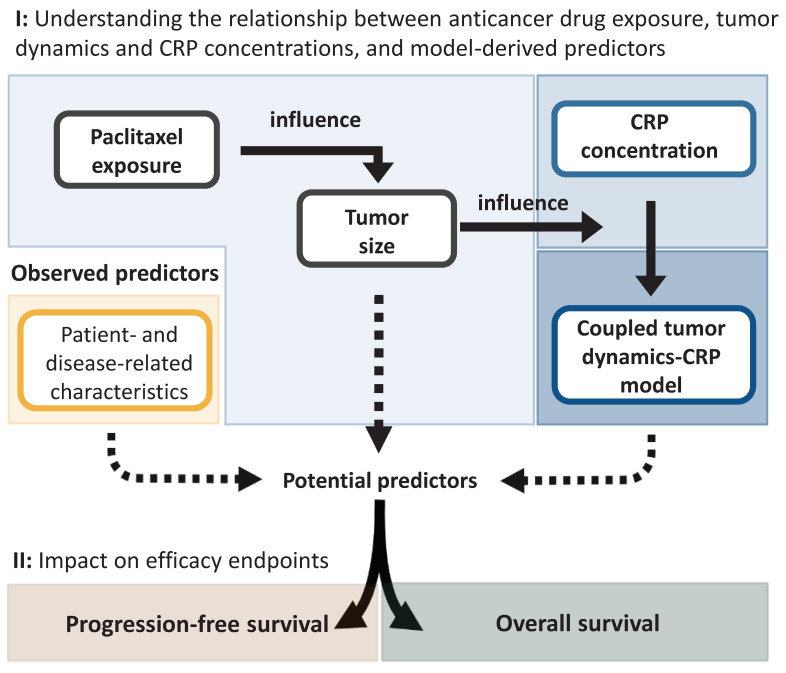
Schematic overview of the different stages undertaken to identify significant predictors of progression-free survival and overall survival. CRP: C-reactive protein.

The developed modeling framework comprised two stages. First, in order to establish the quantitative relationship between anticancer drug exposure, tumor dynamics, and CRP concentration, a TS model was developed to characterize the change in TS over time while accounting for the influence of drug exposure. Model-predicted longitudinal TS (as a metric of tumor dynamics) was then coupled to a CRP model to characterize circulating CRP concentrations using nonlinear mixed-effects models (Figure 1, I). In the second stage, patient- and disease-related characteristics as well as different model-derived metrics of CRP and TS were explored as predictors of PFS and OS by means of a parametric time-to-event model (Figure 1, II).

#### 2.2.1. Characterization of the Relationship between Drug Exposure, Tumor Dynamics, and C-Reactive Protein Concentration

##### Characterization of Tumor Dynamics

A recently published TS model based on the presented clinical data was used to characterize the time course of TS and predict individual TS over time (Figure 1, I). The detailed development of the TS model has been previously published [15]. In brief, change in TS over time was described considering a linear net tumor growth and first-order drug-induced tumor decay. Drug-induced tumor decay was described as a function of paclitaxel area under the plasma concentration-time curve from the start to end of a cycle (AUC_cycle_), based on a single paclitaxel dose, administered on the first day of the 21-day cycle. To account for the development of drug resistance, the drug effect was estimated as exponentially decreasing over time (the equation describing the TS model is provided in Supplementary Section S1a). 

Model development was initially based only on the PK-guided dosing arm data due to non-availability of paclitaxel PK data in the BSA-guided dosing arm but was later extended to include both the BSA- and PK-guided dosing arms using the multiple imputation approach [15,27,28,29]. 

Population TS model parameters and their uncertainty were derived from the multiple imputation framework as described by Rubin [15,27,30]. Additionally, individual TS parameter estimates were derived following the same principle described by Rubin [30] (further details in Supplementary Section S1b). This allowed for a sequential modeling approach where individual TS parameter estimates along with their uncertainty would be leveraged to estimate TS over time and consequently allowed to influence CRP production [31]. 

##### Characterization of C-Reactive Protein Concentration-Time Course

The CRP model aimed to characterize circulating CRP concentrations through a turnover model: in this model, CRP concentrations were governed by a zero-order production with rate constant $K_{in}$ and a first-order degradation with rate constant $K_{out}$ [32,33]. For baseline CRP concentration, a steady state was assumed corresponding to the ratio of $K_{in}$:$K_{out}$ [32,33]. $K_{out}$ was fixed to result in a CRP half-life of 19 h, which was reported to be independent of the (patho)physiological state of the individual [34]. Only interindividual variability (IIV) associated with $K_{in}$ was considered, since it was the single determinant of CRP concentration. Moreover, additional residual variability that could not be explained by IIV was also quantified. 

Variables that could impact CRP concentrations were chosen based on clinical relevance and graphical correlations (i.e., correlation between those variables and individual $K_{in}$) and were investigated for a potential statistically significant impact only on $K_{in}$—as the precursor conversion step of CRP production—using a stepwise covariate model (SCM)-building approach [35,36] (further details on the model development, list of tested variables, and description of the SCM procedure are provided in Supplementary Sections S1c and d).

##### Linking Tumor Dynamics to C-Reactive Protein Concentration-Time Course

To simultaneously associate the estimated individual TS to CRP production (Figure 1, I), different models were explored. These included assuming a direct influence (linear, exponential, power, fractional change models) as well as investigating the potential for a delayed effect (effect compartment, different transit compartments with *n* = 1–4 compartments). Moreover, different TS metrics were explored: absolute TS, the difference in TS from baseline TS, the ratio of TS to baseline TS (i.e., fold change in TS from baseline TS), and TS slope (i.e., first derivative over time). A combined influence of baseline TS and the difference in TS from baseline TS, or the relative change in TS to baseline TS, was also investigated. 

In a subsequent step, to account for the heterogeneity in the patients’ CRP profiles and the diverse individual TS, the impact of accounting for IIV on the parameters of the function describing the relation between the TS metric and CRP production was explored. 

Selection and evaluation of the best model for the characterization of circulating CRP concentration was based on parameter precision, numerical improvement in model performance, goodness-of-fit (GOF) plots, and predictive performance demonstrated by the visual predictive checks (VPC) for models that successfully converged. Moreover, model robustness and parameter uncertainty were assessed through bootstrapping the dataset 1000 times. 

#### 2.2.2. Characterization of Efficacy Endpoints and Their Predictors

Clinical efficacy endpoints (PFS and OS) were characterized to evaluate the prognostic value of the different predictors using parametric time-to-event (TTE) models, and later to allow for simulations under different predictor levels. Different hazard functions (exponential, Weibull, Gompertz, log-logistic, and lognormal) were explored to characterize PFS events over time. For OS, exponential, Weibull, and Gompertz hazard functions were explored. Since it was previously demonstrated that no significant difference in PFS or OS existed between both treatment arms (*p*-value = 0.228 and *p*-value = 0.682, respectively [22]), no stratification was adopted, and both arms were pooled for a joint characterization of PFS and OS. No variability (i.e., IIV) was considered, as only one event was available per patient.

Since the primary objective was to identify *early* longitudinal predictors of PFS and OS, we focused on metrics derived from the first three treatment cycles. Consequently, a landmark time [37] was chosen at the beginning of treatment cycle 3 (i.e., day 42 from the start of treatment) such that PFS would be defined as the time from the start of treatment cycle 3 until objective tumor progression or death, whichever occurs first, and OS would be defined as the time from the start of treatment cycle 3 until patient death. Table 1 lists the model-derived CRP- and TS-related metrics calculated from the early (< treatment cycle 3) longitudinal CRP and TS predictions, respectively as well as neutrophil-to-lymphocyte ratio-related metrics. In addition, factors relating to disease aggressiveness and overall health status of the patient were also considered and included: baseline Eastern Cooperative Oncology Group (ECOG) performance status, smoking status, presence/absence of liver lesions, presence/absence of brain lesions, disease stage, tumor histology, number of target lesions, number of non-target lesions, and sum of target and non-target lesions.

Predictors were tested on the hazard function that best described the distribution of the events using an SCM approach, with the same statistical criteria as described in Supplementary Section S1d. In addition to these criteria, the precision of the estimates of the parameter and predictor effect, and the absence of correlation to a previously included predictor were further criteria for the inclusion of a predictor.

Comparison and selection of the different TTE models were based on the precision of parameter estimates, numerical improvement (calculated using the Akaike information criterion, AIC), and the model’s predictive performance based on Kaplan-Meier VPC (KM VPC) where proportions of the predicted patients not having an event were plotted along with their 90% CI against the proportion of the observed events in the patients. Then, both the observed and median distributions of the TTE were compared. The robustness of the model and the uncertainty of the final model parameter estimates were assessed through a bootstrap (*n* = 1000).

#### 2.2.3. Assessment of the Impact of Identified Predictors of Efficacy

The impact of the identified significant predictors on the event of interest (PFS, OS) was quantified through simulations (*n* = 250) using the final TTE model and the different percentiles of the distribution of each of the significant continuous predictors or the different categories, in the case of a categorical predictor. The impact on median TTE could then be assessed and compared to the observed median TTE.

### 2.3. Data Analysis and Software

All modeling and simulation work was performed with NONMEM 7.4.3 (Icon Development Solutions, Ellicott City, MD, USA) [38] using PsN 4.8.1 (Uppsala University, Uppsala, Sweden) [36] and Piraña 2.9.4 (Certara Inc., Princeton, NJ, USA) [39]. Dataset preparation and visualizations were performed in R 3.5.3 (The project for statistical computing. Vienna, Austria) [40]. Parameters were estimated using the first-order conditional estimation method with interaction in the tumor dynamics and CRP models, while in the TTE models, parameters were estimated with the first-order method since one observation per patient was available, and no IIV was estimated. 

## 3. Results

### 3.1. Clinical Data

**Figure 2:**
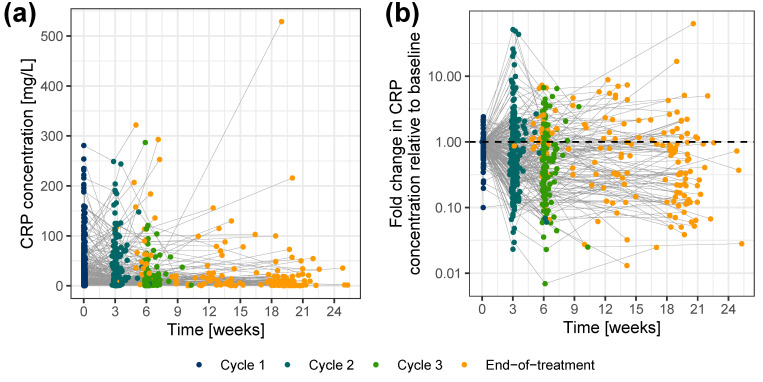
CRP concentrations versus time (**a**) on a linear scale, and (**b**) as fold change in CRP concentrations relative to baseline, color-coded by sample time. Colored dots: CRP concentrations; gray lines: connected data points per individual. CRP: C-reactive protein.

Out of the 365 patients enrolled in the CEPAC-TDM study, 258 patients were enrolled in the biomarker substudy and had longitudinal CRP concentrations totaling 945 CRP measurements. The demographic and clinical characteristics of these 258 patients (Supplementary Table S2) were similar to the full patient population [22]. Six CRP samples (0.635%) were below the lower limit of quantification (LLOQ), and only one CRP sample (0.106%) was above the upper limit of quantification (ULOQ). Because of the low percentage of below LLOQ samples, they were excluded from the analysis dataset with the assumption that they would not significantly influence model development. The ULOQ sample, which was subjected to a dilution validation, was temporarily excluded during the model development stages to avoid potential bias but was later re-introduced at the final stage of each key model. Only one patient had two CRP measurements, both of which were below LLOQ, and thus that patient was excluded from the analysis. Hence, the analysis dataset summed up to a total of 257 patients (939 CRP measurements: median 13.4 mg/L; range 0.320–529 mg/L). The sampling frequency of CRP is represented in supplementary Table S3, and the CRP concentration-time profile is represented in Figure 2. Details of TS measurements have been previously reported [15]. Times of disease progression and death were available for 58.5% and 67.8% of the patients, respectively. Patients with unknown progression or survival time were right censored at their time of last observation. The maximum follow-up time for progression was 28.7 months and for survival was 32.5 months. Median PFS and OS were 5.97 months and 10.3 months, respectively.

### 3.2. Modeling Framework

#### 3.2.1. Characterization of the Relationship between Drug Exposure, Tumor Dynamics, and C-Reactive Protein Concentration 

##### Characterization of Tumor Dynamics

The median values of our derived individual TS parameter estimates were in line with the final population TS parameter estimates from both treatment arms previously reported in [15] (Supplementary Table S4). Net tumor growth was estimated to occur linearly at a rate of 0.742 mm/month. Paclitaxel-induced tumor decay occurred at a first-order rate of 2.30 × 10^−5^ (µmol/L·h)^−1^·h^−1^, whereas baseline paclitaxel effect declined at an exponential rate of 0.021 day^−1^, resulting in a 58.6% (46.1–68.3%) reduced drug effect at the end-of-treatment cycle 2 [15]. A detailed description of the TS model performance and its evaluation has been previously published [15]. 

##### Characterization of C-Reactive Protein Concentration-Time Course

The CRP turnover model predicted both the average and individual CRP concentrations under the steady-state assumption: only baseline IL-6, baseline TS, disease stage, and smoking status affected CRP production, i.e., for a non-smoker with stage IV NSCLC, baseline IL-6 of 2.57 pg/mL and TS of 8.25 cm (median values), $K_{in}$ was estimated to be 0.297 (mg·L^−1^)·h^−1^, corresponding to a population steady-state baseline CRP concentration of 8.14 mg/L (Table 2) [41]. Current smokers, with disease stage IV, high baseline IL-6 (14.9 pg/mL, i.e., 95th percentile), and high tumor load (baseline TS: 17.8 cm, i.e., 95th percentile) would have a 68.3-fold higher $K_{in}$ and consequently a less favorable higher inflammatory level compared to non-smokers with less aggressive disease stage (stage IIIB), lower baseline IL-6 (0.438 pg/mL, i.e., 5th percentile), and lower tumor load (baseline TS: 2.20 cm, i.e., 5th percentile). Functional relationships between $K_{in}$ and these identified variables and the detailed univariate effect of each of these variables on $K_{in}$ are depicted in the Supplementary Section S1e and Figure S3, respectively. The inclusion of these variables explained 27.2% of the IIV associated with $K_{in}$ and resulted in significant model improvement (*p*-value < 0.0001). 

**Figure 3:**
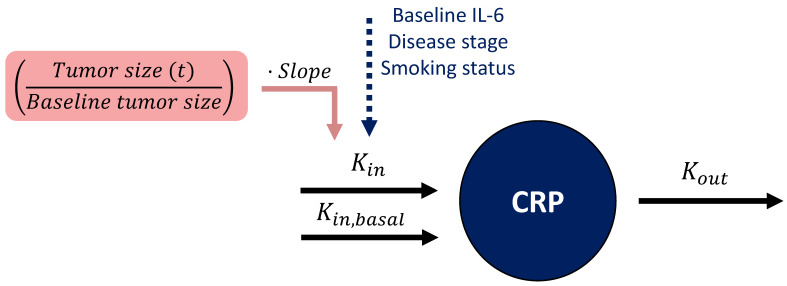
Schematic representation of the coupled tumor dynamics-CRP model. In the CRP turnover model (dark blue), CRP concentration was influenced by two production rate constants ($K_{in}$ ) and ($K_{in,basal}$), the latter being unperturbed by influential variables or tumor size to ensure a basal level of CRP concentration. CRP production rate constant ($K_{in}$) was influenced by baseline interleukin 6 (IL-6), disease stage, and smoking status. The tumor size model-derived longitudinal ratio of tumor size to baseline tumor size (pink) informed CRP production through a linear relationship (i.e., slope parameter). $K_{out}$: CRP degradation rate constant. CRP: C-reactive protein.

Since baseline TS is inherently considered within the longitudinal TS data, the former was removed from the model to avoid its redundant inclusion, before informing the CRP model with longitudinal TS. Additionally, to ensure a basal physiological level of CRP production regardless of the TS impact, a basal unperturbed $K_{in}$ ($K_{in,basal})$ was added, reflecting a basal CRP concentration of 0.3 mg/L (i.e., corresponding to the LLOQ of CRP concentration) (Figure 3).

##### Linking Tumor Dynamics to C-Reactive Protein Concentration-Time Course

The relation between tumor dynamics and CRP concentrations was best characterized by a linear model relating the ratio of TS at any given time to baseline TS (i.e., x-fold change in TS from baseline TS, $(\frac{Tumor\;size\;\left(t\right)}{Baseline\;tumor\;size}$), to CRP production rate constant ($K_{in}$) (Figure 3). For a patient with typical $K_{in}$ = 0.390 (mg·L^−1^)·h^−1^ (Table 2) and a ratio of TS to baseline TS of 1, a change in that ratio from 1 to 0.5 (i.e., 50% tumor shrinkage) would linearly decrease the TS-dependent $K_{in}$ ($K_{in,TS}$) from 0.319 (mg·L^−1^)·h^−1^ to 0.160 (mg·L^−1^)·h^−1^, i.e., a 50% reduction in CRP production rate constant ($K_{in,TS}$ = $K_{in}$·0.819·$\frac{Tumor\;size\;\left(t\right)}{Baseline\;tumor\;size}$). A large IIV of 60.4 CV% was associated with this linear relationship and explained the variable patients’ CRP profiles and their diverse individual TS. Parameters were estimated with good precision (RSE < 26%, Table 2), and a graphical evaluation of the model showed no misspecification and adequate predictive performance (GOF plots and VPC are provided in Supplementary Figures S4 and S5, respectively).

#### 3.2.2. Characterization of Efficacy Endpoints and Their Predictors

##### Progression-Free Survival Model

**Figure 4:**
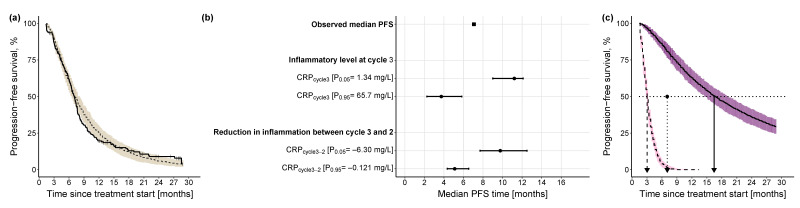
Kaplan-Meier visual predictive checks and impact of identified predictors on median PFS. (**a**) Kaplan-Meier visual predictive checks (*n* = 250) comparing predictive performance of time-to-event final PFS model with lognormal hazard function and identified predictors, to the observed PFS data. Solid line: observed PFS data (thin vertical lines represent censoring times corresponding to the time of the patient’s last participation in the study); dashed line: median model-predicted profile, with 90% confidence interval (beige shade). (**b**) Forest plot of the impact of identified significant predictors on median PFS. Effects of continuous predictors (i.e., CRP_cycle3_, CRP_cycle3-2_) are shown at the 5th and 95th percentiles of the respective predictor. Black dots: predictor effects; horizontal lines: 95% confidence intervals. (**c**) Kaplan-Meier plots of simulated (*n* = 250) PFS profiles under the combined effect of the 5th percentile of the continuous predictors (CRP_cycle3_: 1.34 mg/L, CRP_cycle3-2_: −6.30 mg/L) and the 95th percentile of the continuous predictors (CRP_cycle3_: 65.7 mg/L, CRP_cycle3-2_: −0.121 mg/L). Dashed black line: simulated profile at the 95th percentile predictor level; solid black line: simulated profile at the 5th percentile predictor level; pink shaded area: 90% confidence intervals of simulated profiles at 95th percentile predictor level; purple shaded area: 90% confidence intervals of simulated profiles at 5th percentile predictor level; dotted horizontal line: 50% PFS; dashed vertical line: median PFS time at 95th percentile values of the predictors; dotted vertical line: observed median PFS time; solid vertical line: median PFS time at 5th percentile values of the predictors. CRP: C-reactive protein; PFS: progression-free survival.

Given the chosen landmark time, only patients who survived without progression up to at least treatment cycle 3 (i.e., day 42 from the start of treatment) were included in the TTE analysis (203 out of 257 patients with CRP measurements, 78.9%, median PFS: 7.07 months). An initial increase in hazard followed by a decrease (i.e., a lognormal distribution of the event time described by a parametric lognormal hazard function, Equation (1)) best described the observed PFS over time (Table 3) compared to the other explored hazard functions (detailed comparison against base models with the different hazard functions is provided in Supplementary Section S1f). The inflammatory level at treatment cycle 3, i.e., CRP_cycle3_, and the extent of reduction in the inflammatory level between treatment cycle 3 and cycle 2, i.e., CRP_cycle3-2_, significantly affected the hazard, Equation (2) (i.e., risk of progression or death) (for the derivation of CRP_cycle3_ and CRP_cycle3-2_, see Table 1). Hence, we obtained the following final PFS model:

(1) $$h_{0}\left(t\right)=\frac{1}{t\cdot \sqrt{2\pi \sigma^{2}}}\cdot \frac{e^{\left(-\frac{1}{2}\;\cdot \;{\left(\frac{\log\left(t\right)-\mu }{\sigma }\right)}^{2}\right)}}{1-\phi \;\left(\frac{\log\left(t\right)-\mu }{\sigma }\right)}$$

(2) $$h\left(t\right)=h_{0}\left(t\right)\cdot \left(1+E_{CRP_{cycle3}\;}\cdot CRP_{cycle3}\right)\cdot \;(CRP_{cycle3-2}{}^{E_{CRP_{cycle3-2}}})$$

where $h_{0}\left(t\right)$ is the baseline lognormal hazard function parameterized by the mean (μ) and standard deviation (σ) of the underlying normal distribution, as well as the standard normal cumulative distribution function (ϕ); $E_{CRP_{cycle3}}$ and $E_{CRP_{cycle3-2}}$ are parameters relating the effect of $CRP_{cycle3}$ (linear function) and $CRP_{cycle3-2}\;$ (power function) to the hazard, respectively, while h(t) is the modified lognormal hazard after the inclusion of the identified predictors. Using KM VPC and based on simulations from the final PFS model, Figure 4a shows the predictive model performance after the inclusion of the two CRP-related predictors. Although there was a slight underprediction, i.e., prediction of later PFS events, between 7 months and 14 months, in general, the model adequately predicted the distribution of events over time, and the observed events fell within the 90% CI of the simulated events.

The univariate impact of $CRP_{cycle3}$ and $CRP_{cycle3-2}$ on the median PFS was explored at the upper (95th percentile) and lower values (5th percentile) of their respective distribution (Figure 4b): the inflammatory level at treatment cycle 3 showed a higher impact (i.e., 7.47 months difference between 5th and 95th percentile of $CRP_{cycle3}$) on median PFS compared with the reduction in inflammatory level from treatment cycle 3 to cycle 2 (i.e., 4.66 months difference between 5th and 95th percentile of $CRP_{cycle3-2}$). Additionally, to explore the combined impact of both predictors, for a patient cohort with low inflammatory level at treatment cycle 3 and high reduction in inflammatory level between treatment cycle 3 and cycle 2 (i.e., favorable condition), median PFS was 16.5 months, whereas for a patient cohort with a high inflammatory level at treatment cycle 3 and low reduction in inflammatory level between treatment cycle 3 and cycle 2 (i.e., less favorable condition), median PFS was 13.4 months shorter (Figure 4c).

##### Overall Survival Model

**Figure 5:**
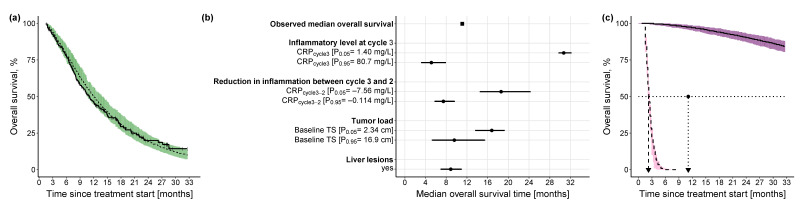
Kaplan-Meier visual predictive checks and impact of identified predictors on median OS. (**a**) Kaplan-Meier visual predictive checks (*n* = 250) comparing predictive performance of time-to-event final OS model with Weibull hazard function and identified predictors to the observed OS data. Solid line: observed OS data (thin vertical lines represent censoring times corresponding to the time of the patient’s last participation in the study); dashed line: median model-predicted profile, with 90% confidence interval (green shade). (**b**) Forest plot of the impact of identified significant predictors on median OS. Effects of continuous predictors (i.e., CRP_cycle3_, CRP_cycle3-2_, baseline tumor size) are shown at the 5th and 95th percentiles of the respective predictor, and effects of categorical predictors (i.e., liver lesions) are shown relative to the reference category. Black dots: predictor effects; horizontal lines: 95% confidence intervals. (**c**) Kaplan-Meier plots of simulated (*n* = 250) OS profiles under the combined effect of the 5th percentile of the continuous predictors (CRP_cycle3_: 1.40 mg/L, CRP_cycle3-2_: −7.56 mg/L, baseline tumor size: 2.34 cm) in absence of liver lesions and the 95th percentile of the continuous predictors (CRP_cycle3_: 80.7 mg/L, CRP_cycle3-2_: −0.114 mg/L, baseline tumor size: 16.9 cm) in presence of liver lesions. Dashed black line: simulated profile at the 95th percentile predictor level in presence of liver lesions; solid black line: simulated profile at the 5th percentile predictor level in absence of liver lesions; pink shaded area: 90% confidence intervals of simulated profiles at 95th percentile predictor level in presence of liver lesions; purple shaded area: 90% confidence intervals of simulated profiles at 5th percentile predictor level in absence of liver lesions; dotted horizontal line: 50% OS; dashed vertical line: median OS time at 95th percentile values of the predictors in presence of liver lesions; dotted vertical line: observed median OS time; solid vertical line: median OS time at 5th percentile value of the predictors in absence of liver lesions. CRP: C-reactive protein; TS: tumor size.

Similar to the PFS TTE model development, only patients who survived up to at least treatment cycle 3 (i.e., day 42 from the start of treatment) were included in the TTE analysis (235 patients out of 257 patients with CRP measurements, 91.4%, median OS: 11.1 months). The observed OS over time was best described by a TTE base model with a parametric Weibull function (Equation (3)) compared with the exponential or Gompertz hazard functions (detailed comparison against base models with the different hazard functions is provided in Supplementary Section S1g). The hazard of death was increasing with time, indicated by the shape parameter (α) estimate of 1.68, i.e., >1 (Table 4). As with PFS, the inflammatory level at treatment cycle 3, i.e., CRP_cycle3_, and the reduction in the inflammatory level between treatment cycle 3 and cycle 2, i.e., CRP_cycle3-2_, significantly affected the hazard (i.e., risk of death). In addition, tumor load, i.e., baseline TS and presence of liver lesions, were identified as significant predictors (Equation (4)). Compared with the base model, the inclusion of these four predictors contributed to an improved model performance, i.e., prediction of the risk of death (*p*-value < 0.0001). Using KM VPC and based on simulations from the final OS model, after the inclusion of these variables, the model demonstrated good predictive performance where the observed events fell within the 90% CI of the simulated events (Figure 5a). Hence, we obtained the following final OS model:

(3) $$h_{0}\left(t\right)=\lambda \cdot \alpha \cdot {\left(\lambda \cdot t\right)}^{\left(\alpha -1\right)}$$

(4) $$h\left(t\right)=h_{0}\left(t\right)\cdot (CRP_{cycle3}{}^{E_{CRP_{cycle3}\;}})\cdot (CRP_{cycle3-2}{}^{E_{CRP_{cycle3-2}}})\cdot \;(BLTS^{E_{BLTS}})\;\cdot \{\begin{array}{c} 1\;\;\;\;\;\;\;\;\;\;\;\;\;\;\;,no\;liver\;lesions\;\\ 1+E_{liver\;},\;with\;liver\;lesions \end{array}$$

where $h_{0}\left(t\right)$ is the baseline Weibull hazard function parameterized by the scale (λ) and shape (α) parameters. $E_{CRP_{cycle3}\;}$, $E_{CRP_{cycle3-2}}$, $E_{BLTS}$, and $E_{liver}$ are parameters relating the effect of $CRP_{cycle3}$ (power function), the difference between $CRP_{cycle3}$ and $CRP_{cycle2}$ ($CRP_{cycle3-2}$, power function), the baseline TS (BLTS, power function), and the presence of liver lesions (fractional change function) to the Weibull hazard, respectively, while h(t) is the modified Weibull hazard after inclusion of these predictors.

The univariate impact of the identified predictors on the median OS was explored at the upper (95th percentile) and lower values (5th percentile) of the respective predictor distribution, in the case of continuous predictors, and in the case of categorical predictors, it was explored with respect to the reference predictor category: the inflammatory level showed by far the highest impact (i.e., 25.6 months difference between the 5th and 95th percentile of $CRP_{cycle3}$) on median OS compared with the reduction in inflammatory level (i.e., 11.15 months difference between the 5th and 95th percentile of $CRP_{cycle3-2}$) or the tumor load (i.e., 7.24 months difference between the 5th and 95th percentile of BLTS) (Figure 5b). For a patient cohort with a low inflammatory level at treatment cycle 3, a high reduction in inflammatory level between treatment cycle 3 and cycle 2, and a low tumor load in the absence of liver lesions, the median OS was not reached until 33 months (Figure 5c), whereas for a patient cohort with a worse-case disease state—a high inflammatory level at treatment cycle 3, a low reduction in inflammatory level between treatment cycle 3 and cycle 2, and a high tumor load in presence of liver lesions—median OS was only 2.24 months (i.e., combined impact of all predictors). 

#### 3.2.3. Impact of Different Levels of Inflammation on Efficacy Endpoints

**Figure 6:**
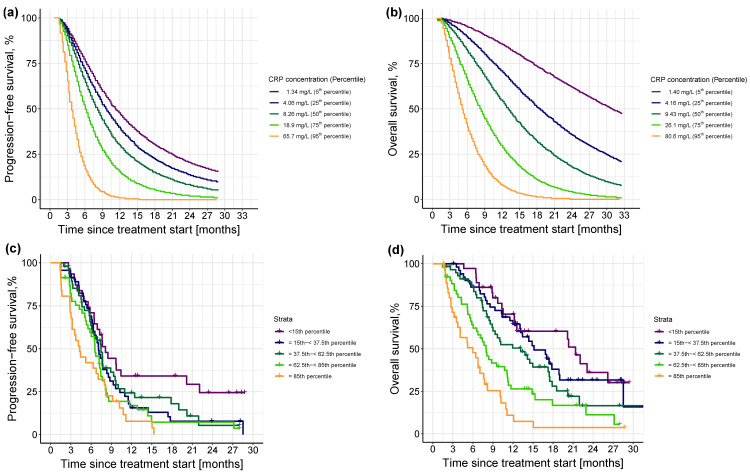
Upper panel: Kaplan-Meier visual predictive checks (*n* = 250) of simulated (**a**) progression-free survival events and (**b**) overall survival events at different concentrations (i.e., percentiles) of CRP_cycle3_. Different colors in the upper panel indicate different percentiles. Lower panel: Kaplan-Meier plots of observed distribution of (**c**) progression-free survival and (**d**) overall survival events stratified by model-estimated CRP_cycle3_, color-coded by the different percentile intervals. Percentile intervals were chosen so that their median corresponds to the percentiles of the simulated profiles in the upper panel, i.e., 5th, 25th, 50th, 75th, and 95th. Solid colored line: observed progression-free survival or overall survival; thin vertical lines: censoring times corresponding to the time of the patient’s last participation in the study; different colors indicate different percentile intervals.

Since the inflammatory level at treatment cycle 3 was the most impactful predictor of both PFS and OS, the prognostic value of the different levels of $CRP_{cycle3}$ were systematically explored. Simulations from the final PFS and OS models based on the different percentiles of $CRP_{cycle3}$ showed an even impact on PFS and OS, with—as expected—a shorter PFS and OS associated with an increased inflammatory level, and no threshold detected (Figure 6a,b, respectively). Comparison of these simulated profiles against the observed PFS and OS events and based on different percentile intervals of the model-estimated $CRP_{cycle3}$ is shown in Figure 6c,d, respectively. In the PFS KM plot (Figure 6c), the upper and lower percentile intervals showed a similar pattern to the simulated profiles (Figure 6a) and the lowest three percentile intervals showed a comparable median PFS, although there was an observed overlap of the intermediate$\;CRP_{cycle3}$ percentile intervals. For OS, Figure 6d depicted a similar pattern to the simulation results (Figure 6b) with a corresponding median OS for all the percentile intervals—except for the lowest two which were simulated to have a longer median OS.

## 4. Discussion

We successfully identified early predictors of efficacy in advanced NSCLC patients and demonstrated that not only lower concentrations of CRP_cycle3_ (i.e., lower inflammatory state) but also a larger reduction in the inflammatory level, specifically a larger difference between CRP_cycle3_ and CRP_cycle2_ (i.e., 5th percentiles), were associated with longer PFS (median PFS: 16.5 months; observed median PFS: 7.07 months). In addition to the latter two CRP-related metrics, lower tumor load (i.e., baseline TS, 5th percentile), and absence of liver lesions were associated with longer survival (median OS not reached after 33 months; observed median OS: 11.1 months). The developed coupled tumor dynamics-biomarker model predicted circulating CRP concentrations and characterized the association between TS, a metric of tumor dynamics, and CRP, an inflammatory serum biomarker and a marker of disease aggressiveness. Moreover, this coupled model paved the way to explore the predictive value of model-derived variables of the longitudinally predicted CRP and TS data, along with patient- and disease-related characteristics with respect to PFS and OS in patients with advanced NSCLC. 

We leveraged our previously developed TS model [15], where tumor decay was linked to paclitaxel PK exposure to account for dose adaptations. We adopted a sequential modeling approach [31] where individual TS parameter estimates along with their uncertainty were leveraged to estimate TS over time and consequently informed circulating CRP concentrations. This approach was chosen for its higher precision and lower bias compared to only using the population parameter estimates [31], and to account for the variability and uncertainty encountered from the multiple imputation framework within which the TS model was developed. A similar framework, linking tumor dynamics to the circulating serum biomarkers, was previously applied in small-cell lung cancer [42]. In that framework, a hypothetical tumor dynamic compartment, informed by the potential impact of the different treatment strategies influenced the biomarker concentrations; however, no impact of the drug concentration was considered. Thus, to our knowledge, our developed modeling framework is the first to link drug exposure and tumor dynamics to serum biomarker concentration in NSCLC, which we believe can be applied in different settings within oncology, e.g., different treatment modalities and/or cancer types. 

The identified influential variables on CRP production were plausible, being baseline IL-6, baseline TS, disease stage, and smoking status. IL-6 is the biochemical precursor to CRP [10], whereas baseline disease stage and TS are metrics of disease aggressiveness and reflect CRP’s positive correlation with advanced disease stage and metastasis [11]. Smoking status was previously confirmed to have a positive impact on CRP concentration, even after smoking cessation, possibly mediated through a chronic systemic inflammatory response and oxidative stress [43,44]. Thus, a current smoker with high inflammatory cytokine concentration, i.e., baseline IL-6, high tumor burden, and more advanced disease stage, would have a 68.3-fold faster CRP production rate and consequently a higher, unfavorable inflammatory level than a non-smoker with low inflammatory response, low tumor burden, and less advanced disease stage.

The association between CRP and NSCLC is not fully clear yet; however, IL-6 and the inflammatory state are expected to play a role [11,45]. Different mechanisms have been postulated for the relation between tumor and CRP: (a) CRP could be released in response to the inflammatory status caused by the tumor growth, (b) CRP could be a result of the body’s response to tumor antigens or the increased production of inflammatory cells from the malignant cells [12], or (c) CRP release is mediated through its precursor IL-6 which has been shown to be upregulated in response to treatment, e.g., paclitaxel and platinum drugs and involved in cancer resistance [45]. The linear model linking the TS relative to baseline TS, to CRP production translates to the fact that the inflammatory status associated with the malignant tumor stimulates inflammatory cytokines that in turn activate the hepatocytes to release CRP [11]. 

Our coupled tumor dynamics-CRP model predicted TS and CRP concentrations across the whole duration of treatment. Hence, post-treatment TS and CRP concentrations were estimated for all patients and allowed for a rich assessment of non-baseline CRP concentrations and tumor shrinkage at later time points. Nevertheless, our focus was on “early” metrics for future translation into clinical practice where early metrics are necessary for timely decision-making. For this reason, we identified the first three treatment cycles to be of relevance and consequently chose a landmark time defined at the start of treatment cycle 3 for investigation of PFS and OS and did not explore later predictors or the impact of later landmark times. Landmark survival analysis has been commonly applied before [46,47,48,49] to investigate non-baseline predictors, e.g., tumor dynamics, and avoids predicting events before the monitoring time of the predictor (i.e., prediction of PFS or OS before assessment of CRP at treatment cycle 3) [20].

Characterization of PFS and OS by means of a parametric TTE model with the chosen landmark time demonstrated an initial increase in the risk of PFS followed by a later decrease (i.e., lognormal distribution of hazard), probably initially dominated by progression and death events and later dominated only by the death events, whereas a Weibull distribution of hazard characterized the increasing risk of death with time. While previous work has already reported a positive correlation between baseline CRP concentrations and poor prognosis in NSCLC [13,15,45,50], no one, to our knowledge, has looked into CRP kinetics over the treatment time. Exploration of potential predictors revealed superior predictivity for non-baseline CRP concentrations compared with baseline CRP concentrations. Specifically, CRP_cycle3_ was a stronger predictor for both PFS and OS compared with the other time points, e.g., baseline CRP or CRP_cycle2_. This indicated that as the patient moves further in time (i.e., treatment cycles), CRP becomes more representative of the patient’s disease status especially after receiving treatment, compared with baseline measurement that only reflects the patient’s situation before treatment starts. Additionally, the degree of reduction in the inflammatory level between treatment cycle 3 and cycle 2 (CRP_cycle3-2_) was also a strong predictor of PFS and OS, adding to the fact that not only absolute CRP concentration was reflective of the patient’s prognosis but also its dynamic change and reduction across treatment duration. 

CRP at later treatment cycles was always a dominating significant predictor. An investigation of a landmark time at treatment cycle 2, compared to our presented results at a landmark time at treatment cycle 3, identified very similar predictors but with a weaker impact. The most recent CRP concentration, i.e., CRP_cycle2_, was the most significant predictor of PFS, and along with liver lesions, of OS (for OS, BLTS was significant at *p*-value 0.05 but not 0.01, and CRP_cycle3-2_ was no longer an investigated variable). This indicated that the later CRP time points were always the most statistically significant predictors in comparison to baseline measurements. Whereas on the one hand, a landmark time at treatment cycle 3 offers the opportunity to identify more impactful predictors and account for the extent of reduction in CRP concentrations, an even earlier monitoring time—if needed—still identified CRP to be dominating and CRP_cycle2_ to be the strongest predictor of efficacy endpoints, despite a weaker magnitude of impact compared with CRP_cycle3_ (parameter effects for CRP_cycle2_ and CRP_cycle3_ on PFS hazard function 0.0416 vs. 0.109 and on OS hazard function 0.304 vs. 0.781, for landmark times at treatment cycles 2 and 3, respectively).

In addition to CRP-related metrics, the presence of liver lesions and a higher tumor load were associated with shorter survival, i.e., worse prognosis. Our results are in line with previous work [51,52,53], which reported worse prognosis for lung cancer patients with liver metastasis, whereas, similarly, baseline TS was a consistent prognostic variable in NCSLC patients [15,46,54]. Although tumor shrinkage was previously reported to have a significant impact on efficacy outcomes [46,47,55,56,57,58], in our work, only baseline TS, as a tumor-related metric, was significant for OS. This could be attributed to the fact that in the previous work, only baseline characteristics were explored besides the longitudinal tumor-size changes, whereas in our presented work, longitudinal CRP- and neutrophil-to-lymphocyte ratio-related metrics were additionally explored that could have masked the impact of tumor shrinkage. This is a sign that longitudinal variables, whenever present, dominate their respective baseline variables and that longitudinal CRP is more significant than longitudinal TS, when tested combined. Finally, we also demonstrated the profound impact the inflammatory status has, as depicted by the different CRP_cycle3_ concentrations, on the risk of PFS and OS. Thus presenting a promising prognostic marker of disease outcome if monitored through measuring CRP concentration. It is worth noting that we focused on whether monitoring inflammation, through longitudinal CRP measurements, could reflect disease outcome rather than whether modulating inflammation could be a good prognostic factor. Therefore, the impact of IL-6 modulators/blockers and/or reduction in CRP was not our intended objective. On the contrary, we sought to leverage the elevated inflammatory markers as a reflection of the patient’s disease and prognostic status.

Our TTE models did not account for patients who dropped out from the study since according to the study protocol those patients were still followed up for information on progression and survival. Therefore, a drop-out event (i.e., exit from the study) was not considered to be a competing event nor was it considered a censored event on its own. Although this modeling framework was applied to a specific patient cohort and study design, and consequently the impact of the identified predictors is in the first place only applicable to this specific setting, the same modeling framework can generally be applied to explore the potential of different biomarkers and across different treatment modalities within the clinical setting. Given our specific patient cohort and the unique characteristics of our dataset, the evaluation of our model with an external dataset of similar characteristics was not possible. Moreover, splitting the data into training and testing datasets was not an optimal approach given the small number of patients and the risk of loss of power. Nevertheless, to overcome these hurdles, we alternatively sought to compare our simulation-based results to our observed data—as presented in Figure 6. Despite the small number of patients compared to previous assessments [46,52], our developed models precisely estimated the parameters and showed good predictivity and confidence levels. Even though we leveraged baseline IL-6 concentration to inform CRP production, as a significant predictor, the availability of longitudinal IL-6 concentration would have allowed a possible linkage of IL-6 kinetics, as a precursor to CRP production, to the CRP kinetic profile and consequently the possibility for an even earlier prediction of the expected CRP concentration, as previously applied in breast cancer, where IL-6 was found to peak two days before CRP [59]. 

In this work, we aimed to reliably and realistically identify the patients’ expected PFS and OS for relevant actions to be taken, e.g., treatment optimization to tackle the expected outcome. Indeed, it is worth first evaluating this modeling framework for optimal CRP sampling strategies that can provide the most precise predictions for the endpoint of interest in clinical practice. When the most informative timepoints are identified, potential limitations that could arise from longitudinal sampling, e.g., more laborious work, extra cost compared to single sampling, and patient compliance for repeated sampling, are minimized. This could then pave the way for a subsequent step in which the framework could be integrated into an interactive platform, e.g., the R Shiny app for a seamless application in the clinical setting and the real-world of NSCLC. Furthermore, as next steps the impact of different dosing regimens could be investigated or an optimal treatment strategy identified. Thus, this work has the potential to be expanded and linked to dosing recommendations for a model-informed precision dosing and therapy optimization in clinical practice within or outside the scope/therapeutic area of oncology [60,61].

## 5. Conclusions

In conclusion, our clinical data reflected the real-world target population along with the potential and challenges of handling missing data. Our successfully developed tumor dynamics-biomarker model adequately characterized the longitudinal CRP concentrations, providing a link between chemotherapy-driven TS and CRP. Moreover, besides disease-related factors, CRP_cycle3_ and CRP_cycle3-2_ were stronger prognostic factors compared with baseline CRP concentrations to identify patients with advanced NSCLC at earlier risk of progression and/or death, for timely decision-making and therapy optimization. CRP represents a minimally invasive and easily assessed intrinsic serum biomarker. Although it is a non-specific serum biomarker, our work has nevertheless demonstrated that it provides a strong prognostic value regarding patients’ progression and survival, especially when combined with tumor load and liver lesions as in OS. Measuring CRP over the course of treatment and in our case for NSCLC patients at treatment cycle 3 and cycle 2 allows monitoring of the inflammatory level and offers the potential to become a promising prognostic marker to better guide treatment decisions. 

## Figures and Tables

**Table 1:** List of tested predictors in the parametric time-to-event models of progression-free survival and overall survival.

Predictor	Derivation	Abbreviation
**Markers of inflammation**		
CRP-related metrics		
Observed baseline	BLCRP	BLCRP
Model-estimated cycle 1 day 1	CRP_cycle1_	CRP_cycle1_
Model-estimated cycle 2 day 1	CRP_cycle2_	CRP_cycle2_
Model-estimated cycle 3 day 1	CRP_cycle3_	CRP_cycle3_
Absolute difference in CRP concentration:		
cycle 2 from cycle 1	CRP_cycle2_−CRP_cycle1_	CRP_cycle2-1_
cycle 3 from cycle 1	CRP_cycle3_−CRP_cycle1_	CRP_cycle3-1_
cycle 3 from cycle 2	CRP_cycle3_−CRP_cycle2_	CRP_cycle3-2_
Relative change in CRP concentration:		
cycle 2 from cycle 1	(CRP_cycle2_−CRP_cycle1_)/CRP_cycle1_	CRP_(cycle2-1)/cycle1_
cycle 3 from cycle 1	(CRP_cycle3_−CRP_cycle1_)/CRP_cycle1_	CRP_(cycle3-1)/cycle1_
cycle 3 from cycle 2	(CRP_cycle3_−CRP_cycle2_)/CRP_cycle2_	CRP_(cycle3-2)/cycle2_
Fold change in CRP concentration:		
cycle 2 from cycle 1	CRP_cycle2_/CRP_cycle1_	CRP_cycle2/1_
cycle 3 from cycle 1	CRP_cycle3_/CRP_cycle1_	CRP_cycle3/1_
cycle 3 from cycle 2	CRP_cycle3_/CRP_cycle2_	CRP_cycle3/2_
Neutrophil-to-lymphocyte ratio-related metrics		
Observed cycle 1 day 1	N/L_cycle1_	N/L_cycle1_
Observed cycle 2 day 1	N/L_cycle2_	N/L_cycle2_
Absolute difference in neutrophil-to-lymphocyte ratio: cycle 2 from cycle 1	N/L_cycle2_−N/L_cycle1_	N/L_cycle2-1_
Relative change in neutrophil-to-lymphocyte ratio: cycle 2 from cycle 1	(N/L_cycle2_−N/L_cycle1_)/N/L_cycle1_	N/L_(cycle2-1)/cycle1_
Fold change in neutrophil-to-lymphocyte ratio: cycle 2 from cycle 1	(N/L_cycle2_)/(N/L_cycle1_)	N/L_cycle2/1_
**Tumor size-related metrics**		
Observed baseline tumor size	—	BLTS
Model-estimated tumor growth rate	—	$K_{growth}\;$
Model-estimated tumor size at week 7 relative to baseline tumor size	TS_week7_/BLTS	RS7

CRP: C-reactive protein; BLCRP: baseline CRP concentration; TS: tumor size; BLTS: baseline tumor size; TS_week7_: model-estimated tumor size at week 7; RS7: estimated tumor size at week 7 relative to baseline tumor size; N/L: neutrophil-to-lymphocyte ratio; $K_{growth}$: linear net tumor growth rate constant.

**Table 2:** Parameter estimates of the CRP turnover model and the coupled tumor dynamics-CRP turnover model.

Parameter	CRP Turnover Model			Coupled Tumor Dynamics-CRP Turnover Model		
	Estimate	RSE, %	95% CI ^a^	Estimate	RSE, %	95% CI ^b^
Fixed-effect parameters						
$K_{in}$ [(mg·L^−1^)·h^−1^]	0.297	17.9	[0.204, 0.429]	0.390	0.60	[0.252, 0.602]
$K_{in,basal}$ [(mg·L^−1^)·h^−1^]	—	—	—	0.0109 ^c^	—	—
$K_{out}$ [h^−1^]	0.0365 ^d^	—	—	0.0365 ^d^	—	—
Slope (linear parameter linking tumor size to CRP)	—	—	—	0.819	6.70	[0.711, 0.952]
Parameters of the effect of identified variables on $K_{in}$ ^e^						
Baseline IL-6 ^f^	0.263	14.0	[0.175, 0.324]	0.315	8.20	[0.244, 0.363]
Baseline tumor size ^g^	0.0432	28.0	[0.017, 0.070]	—	—	—
Disease stage IIIB relative to stage IV ^h^	−0.401	28.7	[−0.596, −0.102]	−0.392	26	[−0.598, −0.097]
Former smokers relative to non-smokers ^h^	0.536	57.8	[0.020, 1.415]	0.645	12.1	[0.0353, 1.64]
Current smokers relative to non-smokers ^h^	1.11	40.0	[0.378, 2.272]	1.26	19	[0.398, 2.56]
Interindividual variability in respective parameters [CV, %]						
$K_{in}$	95.3	7.60	[80.2, 109]	92.1	7.40	[74.2, 107]
Slope	—	—	—	60.4	15.2	[40.3, 77.9]
$k_{growth}$	—	—	—	100 ^j^	—	—
β	—	—	—	100 ^j^	—	—
λ	—	—	—	100 ^j^	—	—
Baseline tumor size	—	—	—	100 ^j^	—	—
Residual variability						
σ_exp_^i^ [SD, mg/mL]	0.889	3.70	[0.818, 0.953]	0.763	1.70	[0.686, 0.831]

^a^ 95% confidence interval (CI) obtained from 1000 bootstrap runs (successful minimization = 99.6%), ^b^ 95% CI obtained from 1000 bootstrap runs (successful minimization = 97.9%), ^c^ fixed to corresponding lower limit of quantification of CRP concentration (0.3 mg/L), ^d^ fixed to literature value [34], ^e^ for the detailed functional relationships between $K_{in}$ and these identified variables, see Supplementary Section S1e, ^f^ linear function, ^g^ exponential function, ^h^ fractional change function, ^i^ estimated as additive residual variability on log-scale, ^j^ fixed, variability derived from reported uncertainty as described in [31]; β: paclitaxel area under the concentration-time curve from start to end of a cycle-driven tumor decay rate constant at start of treatment (t = 0); CRP: C-reactive protein; CV: coefficient of variation; IL-6: interleukin 6; $K_{in}$: CRP zero-order production rate constant; $K_{in,basal}$: CRP basal unperturbed zero-order production rate constant; $K_{out}$: CRP first-order degradation rate constant; $k_{growth}$: linear net tumor growth rate constant; λ: rate constant for exponential decline in drug effect over time; RSE: relative standard error; SD: standard deviation.

**Table 3:** Parameter estimates of the parametric progression-free survival model ^a^.

Parameter	Estimate	RSE, %	95% CI ^b^
Fixed-effect parameters			
σ [unitless]	0.906	8.80	[0.755, 1.14]
μ [unitless]	9.11	2.50	[8.76, 9.86]
Parameters of the effects of identified predictors on hazard function			
CRP_cycle3_^c^	0.109	55.4	[0.0348, 0.445]
CRP_cycle3-2_^d^	−0.26	37.2	[−0.461, −0.0637]

^a^ time unit of the lognormal hazard function is [hour], ^b^ 95% confidence interval (CI) obtained from 1000 bootstrap runs (successful minimization = 99.9%), ^c^ linear function, ^d^ power function, σ: mean of the lognormal hazard function; μ: standard deviation of the lognormal hazard function; CRP: C-reactive protein; CRP_cycle3_: CRP concentrations at treatment cycle 3; CRP_cycle3-2_: difference in CRP concentrations between treatment cycle 3 and cycle 2; RSE: relative standard error.

**Table 4:** Parameter estimates of the parametric overall survival model.

Parameter	Estimate	RSE, %	95% CI ^a^
Fixed-effect parameters			
λ [1/h]	1.6 × 10^−5^	25.7	[9.32 × 10^−6^, 2.65 × 10^−5^]
α [unitless]	1.68	5.30	[1.54, 1.92]
Parameters of the effects of identified predictors on hazard function			
CRP_cycle3_^b^	0.781	12.8	[0.595, 0.999]
CRP_cycle3-2_^b^	−0.392	24.9	[−0.606, −0.185]
Baseline tumor size ^b^	0.491	33.2	[0.201, 0.881]
Liver lesions ^c^	1.02	36.3	[0.374, 2.03]

^a^ 95% confidence interval (CI) obtained from 1000 bootstrap runs (successful minimization = 98.7%), ^b^ power function, ^c^ fractional change function, α: shape parameter of the Weibull hazard function; λ: scale parameter of the Weibull hazard function; CRP: C-reactive protein; CRP_cycle3_: CRP concentrations at treatment cycle 3; CRP_cycle3-2_: difference in CRP concentrations between treatment cycle 3 and cycle 2; RSE: relative standard error.

## Data Availability

The data generated in this study are not publicly available but will be made available by the corresponding author upon reasonable request. NONMEM model files are available under https://github.com/Kloft-Lab/Nassar-et-al._Tumor_dynamics-CRP-model_NONMEM-scripts (accessed on 9 November 2023).

## Supplementary Materials

The following supporting information can be downloaded at: https://www.mdpi.com/article/10.3390/cancers15225429/s1, Section S1: Supplementary details on the modeling framework (a) characterization of tumor dynamics, (b) derivation of population and individual tumor size model parameters from the tumor size model developed within the multiple imputation framework, (c) characterization of C-reactive protein concentration-time course including Table S1: List of variables tested for impact on C-reactive protein production, (d) stepwise covariate model building, (e) functional relationships between CRP production rate constant ($k_{in})$ and the identified variables, (f) development of time-to-event progression-free survival base model including Figure S1: Kaplan-Meier visual predictive checks comparing predictive performance of time-to-event progression-free survival base models, (g) development of parametric time-to-event overall survival base model including Figure S2: Kaplan-Meier visual predictive checks comparing predictive performance of time-to-event overall survival base models. Section S2: Additional supplementary tables, Table S2: Baseline demographics and clinical characteristics of patients with C-reactive protein measurements, Table S3: Sampling frequency of C-reactive protein, Table S4: Population and individual tumor size model parameters. Section S3: Additional supplementary figures, Figure S3: Forest plot of the impact of significant variables on C-reactive protein production rate constant ($K_{in}$) relative to the reference value, Figure S4: Basic goodness-of-fit plots of coupled tumor dynamics-CRP model, Figure S5: Visual predictive check of the coupled tumor dynamics-CRP model.
