# Supplementary material for: C-Reactive Protein as an Early Predictor of Efficacy in Advanced Non-Small-Cell Lung Cancer Patients: A Tumor Dynamics-Biomarker Modeling Framework
Source: Cancers (Basel). 2023 Nov 15;15(22):5429. doi: 10.3390/cancers15225429 (PMC10670607; doi:10.3390/cancers15225429)
Supplement: Supplementary file 1 [file cancers-15-05429-s001.zip › cancers-2697794-supplementary.pdf]

Supplementary files

1. **Supplementary details on the modeling framework**
2. **Supplementary tables**
3. **Supplementary figures**

1. Supplementary details on the modeling framework

a. Characterization of tumor dynamics

Change in tumor size (TS) over time was described considering a linear net tumor growth (k_{growth}) and first-order drug-induced tumor decay (β_0) as shown in **Eq. 1**. Drug-induced tumor decay was described as a function of paclitaxel area under the plasma concentration-time curve from start to end of a cycle (AUC_{cycle}), based on a single paclitaxel dose administered on the first day of a 21-day cycle. To account for resistance development, the drug effect was estimated as exponentially decreasing over time (λ). Therefore, the change in TS over time was described as follows,

$$\frac{dTS(t)}{dt} = k_{growth}(t) - \beta_0 \cdot e^{-\lambda \cdot t} \cdot AUC_{cycle} \cdot TS(t)$$ **Equation 1**

where $\frac{dTS(t)}{dt}$ denotes the change in TS over time; k_{growth} is the linear net tumor growth rate constant; β_0 is the drug-induced tumor decay rate constant per unit of paclitaxel exposure at time = 0; λ is the rate constant for change in drug effect over time; and AUC_{cycle} is the paclitaxel area under the plasma concentration-time curve from start to end of a cycle based on a single paclitaxel dose administered on the first day of a 21-day cycle.

b. Derivation of population and individual tumor size model parameters from the tumor size model developed within the multiple imputation framework

In contrast to simply taking the arithmetic mean of the individual parameter estimates across the m replicates within the multiple imputation framework, the individual TS parameters along with their associated variances and uncertainty were computed. Hence, the variability and uncertainty associated with the multiple imputation approach were taken into consideration. This allowed for a sequential modeling approach (1) where individual TS parameter estimates along with their uncertainty predict longitudinal TS and consequently influence C-reactive protein (CRP) concentrations.

Derivation of population and individual TS model parameters are outlined in steps 1 and 2, respectively.

Population parameters (step 1)

Population TS model parameter estimates (i.e., k_{growth}, λ, β_0) from the m replicates of the multiple imputation were pooled according to Rubin's rule (**Eqs. 2 and 3**) (2) to obtain the mean of the population parameters (**Eq. 2**) and their associated variances and standard errors (**Eq. 3**).

$$\tilde{\beta} = \frac{1}{m}\sum_{\gamma=1}^{m} \hat{\beta}^{(\gamma)}$$ **Equation 2**

$$\tilde{b} = \frac{1}{m}\sum_{\gamma=1}^{m} \hat{b}^{(\gamma)} + \frac{m+1}{m(m-1)}\sum_{\gamma=1}^{m} (\hat{\beta}^{(\gamma)} - \tilde{\beta})^2$$ **Equation 3**

where in **Eqs. 2 and 3**, m is the number of replicates (here m = 50), β is a TS parameter, $\hat{\beta}^{(\gamma)}$ is the estimate of β from the imputed dataset γ (γ = 1, ..., m), $\tilde{\beta}$ is the calculated estimate of β, $\tilde{b}$ is the calculated variance associated with $\tilde{\beta}$, and $\hat{b}^{(\gamma)}$ is the estimate of b from the imputed data set γ (γ = 1, ..., m).

Individual parameters (step 2)

Following the same principle described by Rubin (2), to obtain individual (i) parameter estimates (i.e., $k_{growth,i}$, λ_i, $\beta_{0,i}$) from the m replicates of the multiple imputation, mean individual parameter estimates, $\tilde{\beta}$, of the individual variability, η , i.e., $\eta_{K_{growth}}, \eta_{\lambda}, \eta_{\beta}$ were computed at an individual level to obtain a single parameter set per individual according to **Eq. 2**. The computed mean individual variability was then transformed to the respective individual parameter estimate as parameterized in the TS model and according to **Eq. 4**,

$$Par_i = Par_{mean} \cdot e^{\eta_{par,i}}$$ **Equation 4**

where Par_i is the individual parameter estimate, Par_{mean} is the mean population parameter estimate obtained from step 1 and $\eta_{par,i}$ is the computed mean individual variability.

The calculated variances, $\tilde{b}$ (**Eq. 3**), associated with the individual TS parameters were therefore the sum of the mean of the individual variances, $\hat{b}$, associated with the individual variability, η; and the second term $\left(\frac{m+1}{m(m-1)}\sum_{\gamma=1}^{m}(\hat{\beta}^{(\gamma)} - \tilde{\beta})^2\right)$ where $\hat{\beta}$ is the estimate of the individual variability, η, and $\tilde{\beta}$ is the calculated mean individual estimate of the individual variability. The variance was afterwards used as the source of uncertainty (standard error) associated with the individual TS parameters.

c. Characterization of C-reactive protein concentration-time course

The CRP model aimed to characterize circulating CRP concentrations through a structural turnover model. In this model, CRP concentration was governed by a zero-order production rate constant (K_{in}) and a first-order degradation rate constant (K_{out}) (3,4). Initially, a steady state was assumed, where baseline was derived from the ratio of K_{in}:K_{out} (3,4). K_{out} was fixed to result in a CRP half-life of 19 h ($K_{out} = \frac{ln2}{19\,h}$), which was reported to be independent of the (patho)physiological state of the individual (5).

As K_{in} was the single determinant of CRP concentration and the only estimable parameter, interindividual variability (IIV) was considered only on K_{in} as an exponential function assuming a lognormal distribution. Residual variability was modeled as an additive component in the log domain (corresponding to an exponential relation in the linear scale).

Variables that could impact CRP concentrations were chosen based on clinical relevance and graphical relations (i.e., correlation between those variables and K_{in}, **Table S1**) and were investigated for a potential statistically significant impact only on K_{in} —as the precursor conversion step of CRP production—using a stepwise covariate model (SCM)-building approach (6).

Table S1. List of variables tested for impact on C-reactive protein production

Characteristics of variables	Continuous variables	Categorical variables
**Variables that reflect disease aggressiveness**	• Baseline tumor size	• Disease stage<br>• Baseline ECOG status<br>• Presence/absence of brain lesions<br>• Number of target lesions<br>• Number of non-target lesions<br>• Sum of target and non-target lesions
**Variables that reflect an inflammatory status**	• BMI as marker of obesity	• Smoking status
**Variables of physiological relevance to CRP production by hepatocytes**	• ALT and AST as markers of liver injury<br>• Baseline IL-6 as cytokine precursor of CRP production	• Presence/absence of liver lesions as marker of liver injury
**Variables showing correlation with CRP production in the graphical exploration**	—	• NSCLC histology

ALT: alanine amino transferase activity; AST: aspartate amino transferase activity; BMI: body mass index; CRP: C-reactive protein; ECOG: Eastern Cooperative Oncology Group; IL-6: interleukin 6; NSCLC: non-small-cell lung cancer.

d. Stepwise covariate model building

To identify the most statistically significant variables impacting specific model parameter(s), the SCM building approach provided by PsN (6,7) was used.

The SCM was a two-step procedure. In the first step, different variables were included univariately to the parameter and the variable that resulted in the most significant improvement in model performance (i.e., lowest p-value) was retained in the model. The process was repeated with the remaining variables until no further inclusion of a parameter-variable relationship resulted in a significant model improvement (*full model*).

In the second step, starting with the full model, a stepwise backward elimination step was performed where each of the included variables was removed, one at a time, and the variable that did not result in a significant worsening in the model performance was excluded. This continued until removal of any variable was associated with significant worsening of model performance (6).

In this work, a *p*-value <0.05 per 1 df was set as a threshold for inclusion of a parameter-variable relationship in the forward inclusion step and a stricter criterion of *p*-value <0.01 per 1 df was set as a threshold for the exclusion of a parameter-variable relationship in the backward elimination step, to retain variables of highest impact in the *final model*.

e. Functional relationships between CRP production rate constant (k_{in}) and the identified variables

The functional relationships between k_{in} and the identified variables interleukin-6 (IL6), baseline TS (BLTS), smoking status, and disease stage are described in **Eq. 5**,

Equation 5

$$k_{in} = \theta_{Kin} \cdot \left(1 + \theta_{IL6} \cdot (IL6 - 2.57)\right) \cdot \exp\left(\theta_{TS} \cdot (BLTS - 8.25)\right)$$

$$\cdot \begin{cases} 1 & , Non-smokers \\ 1 + \theta_{SMK2} & , Former\ smokers \\ 1 + \theta_{SMK3} & , Current\ smokers \end{cases}$$

$$\cdot \begin{cases} 1 & , Stage\ IV \\ 1 + \theta_{stage} & , Stage\ IIIB \end{cases} \cdot \exp(\eta_{Kin})$$

where θ_{Kin} is k_{in} of a non-smoker with disease stage IV, baseline IL6 of 2.57 pg/L, and baseline TS of 8.25 cm (median values); θ_{IL6} is the fractional change in CRP production rate constant per unit change in baseline IL-6 from the median value of 2.57 pg/L; θ_{TS} is the exponent reflecting the change in the natural log k_{in} per unit change in baseline tumor size from the median value of 8.25 cm; θ_{SMK2} and θ_{SMK3} are the fractional changes in k_{in} in former and current smokers, respectively, compared with non-smokers; and θ_{stage} is the fractional change in k_{in} in patients with disease stage IIIB compared with patients with disease stage IV.

f. Development of time-to-event progression-free survival base model

A landmark survival analysis with a landmark time chosen at the beginning of treatment cycle 3 (i.e., day 42 from the start of treatment) was performed. The TTE base model with a parametric lognormal function (**Figure S1, E**) best described the observed progression-free survival (PFS) events over time compared with the exponential (**Figure S1, A**), Weibull (**Figure S1, B**), Gompertz (**Figure S1, C**) or log-logistic (**Figure S1, D**) hazard functions, where all showed a strong underprediction of PFS events after 6 months. Even though the lognormal hazard function slightly underpredicted PFS events between 7 months and 14 months, they were still closely aligned within the 90% confidence interval (shaded beige area) (**Figure S1, E**). Moreover, the lognormal hazard function was associated with superior model performance compared with the exponential (ΔAIC= +60.6), Weibull (ΔAIC= +37.8), Gompertz (ΔAIC= +60.5), or log-logistic (ΔAIC= +36.6) hazard functions.

Therefore, the lognormal TTE model was adopted as the base model for the subsequent exploration of predictors.

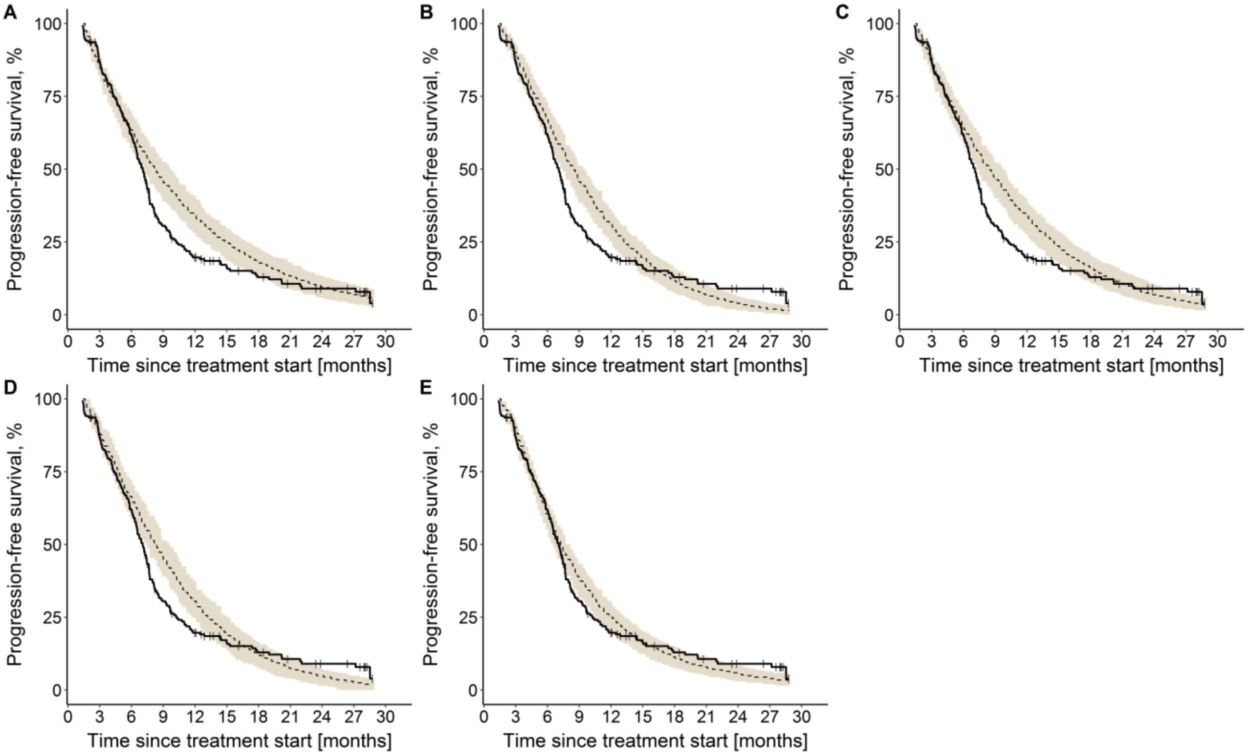

Figure S1. Kaplan-Meier visual predictive checks (n = 250) comparing the predictive performance of time-to-event base models with (A) exponential, (B) Weibull, (C) Gompertz, (D) log-logistic, and (E) lognormal hazard functions to the observed progression-free survival data.
Solid line: observed progression-free survival data (thin vertical lines represent censoring times corresponding to the time of the patient's last participation in the study), dashed line: median model predicted profile, with 90% confidence interval (beige shade).

g. Development of parametric time-to-event overall survival base model

Analogous to the PFS time-to-event anlaysis, a landmark survival analysis with a landmark time chosen at the beginning of treatment cycle 3 (i.e., day 42 from the start of treatment) was performed. The TTE base model with a parametric Weibull function best described the observed overall survival over time compared to the exponential and Gompertz hazard functions (**Figure S2**). Despite overpredicting survival between 6 months and 17 months (i.e., underprediction of the hazard of death), and a slight underprediction after 24 months, the Weibull TTE model still showed an adequate description in the early months (<6 months) and between 17 and 24 months (**Figure S2, B**). On the other hand, although the exponential and Gompertz TTE models adequately predicted the risk of death during the first 6 months (**Figure S2, A, C**), the constant TTE model showed a consistent overprediction of survival over the entire time-course (**Figure S2, A**), whereas the Gompertz TTE model alternated between an overprediction of survival (between 6 months and 24 months) followed by an underprediction (>24 months) (**Figure S2, C**). Moreover, they did not provide a significant improvement in model performance compared with the Weibull TTE model (ΔAIC= +20.1 and +16.1, respectively).

Therefore, the Weibull TTE model was adopted as the base model for the subsequent exploration of predictors.

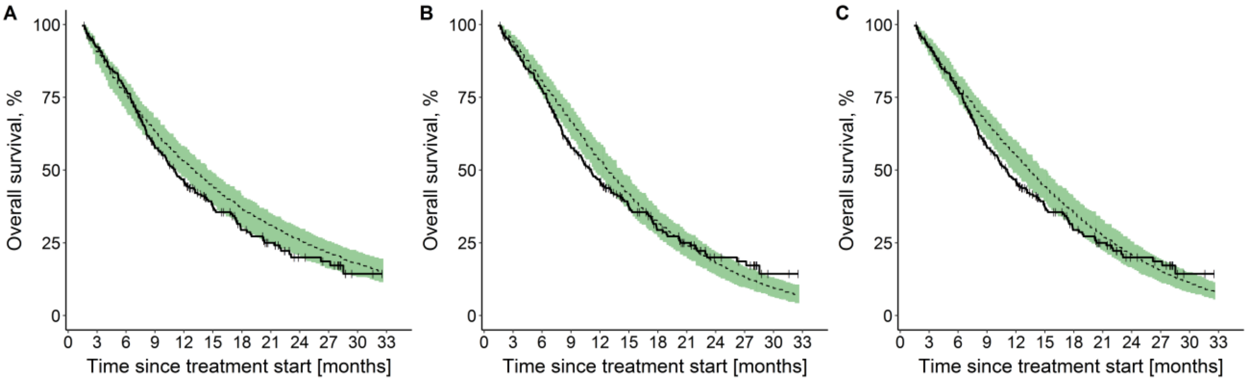

Figure S2. Kaplan-Meier visual predictive checks (n = 250) comparing the predictive performance of time-to-event base models with (A) exponential, (B) Weibull, and (C) Gompertz hazard functions to the observed overall survival data.
Solid line: observed survival data (thin vertical lines represent censoring times corresponding to the time of the patient's last participation in the study), dashed line: median model predicted profile, with 90% confidence interval (green shade).

2. Additional supplementary tables

Table S2. Baseline demographics and clinical characteristics of patients with C-reactive protein measurements

Patient characteristic	All patients (n=258)
Age [years]	
median [range]	64.0 [41.0, 78.0]
Sex, n (%)	
Female	91 (35.3)
Male	167 (64.7)
Treatment arm, n (%)	
BSA-guided paclitaxel dosing arm	126 (48.8)
PK-guided paclitaxel dosing arm	132 (51.2)
Body weight [kg]	
median [range]	74.0 [42.0, 135]
Body height [cm]	
median [range]	171 [146, 194]
Body mass index [kg/m^2]	
median [range]	24.9 [16.8, 41.7]
Body surface area [m^2]	
median [range]	1.86 [1.34, 2.49]
Alanine amino transferase activity [U/L]	
median [range]	23.0 [5.00, 125]
Aspartate amino transferase activity [U/L]	
median [range]	21.0 [9.00, 212]
Smoking status, n (%)	
Non-smokers	28 (10.9)
Former smokers	132 (51.2)
Current smokers	98 (38.0)
Disease stage, n (%)	
IIIB	41 (15.9)
IV	217 (84.1)
NSCLC histology, n (%)	
Adenocarcinoma	165 (64.0)
Squamous-cell carcinoma	60 (23.3)
Bronchioalveolar carcinoma	1 (0.388)
Carcinoma, not otherwise specified	32 (12.4)
Brain lesions, n (%)	
No	224 (86.8)
Yes	34 (13.2)
Liver lesions, n (%)	
No	207 (80.2)
Yes	51 (19.8)
Baseline ECOG performance status, n (%)	
0	135 (52.3)
1	105 (40.7)
2	18 (6.98)

BSA: body surface area; ECOG: Eastern Cooperative Oncology Group; NSCLC: non-small-cell lung cancer; PK: pharmacokinetic.

Table S3. Sampling frequency of C-reactive protein

Time of C-reactive protein sample	Number of samples	
	Day 1	Day 2
Cycle* 1	255	87
Cycle* 2	210	70
Cycle* 3	147	—
End-of-treatment	170	—
**Total**	**939**	

*Each cycle was 3-week long

Table S4. Population and individual tumor size model parameters

Parameter [unit]	Population parameters*		Individual parameters	
	Parameter estimate	RSE, %	Parameter estimate median [range]	SE, % median [range]
k_{growth} [cm/h]	1.03×10^{-4}	43.2	1.04×10^{-4} [2.79×10^{-5}, 3.88×10^{-3}]	1.17 [0,1.25]
β [(µmol/L·h)$^{-1}$·h^{-1}]	2.30×10^{-5}	12.8	2.30×10^{-5} [1.11×10^{-5}, 2.11×10^{-4}]	0.612 [0,0.853]
λ [1/h]	8.75×10^{-4}	15.4	8.75×10^{-4} [2.42×10^{-4}, 1.22×10^{-3}]	0.494 [0,0.538]

*As reported in (8)

β: paclitaxel area under the plasma concentration-time curve from start to end of a cycle-driven tumor decay rate constant at start of treatment (time = 0); k_{growth}: linear net tumor growth rate constant; λ: rate constant for exponential decline in drug effect over time; RSE: relative standard error; SE: standard error.

3. Additional supplementary figures

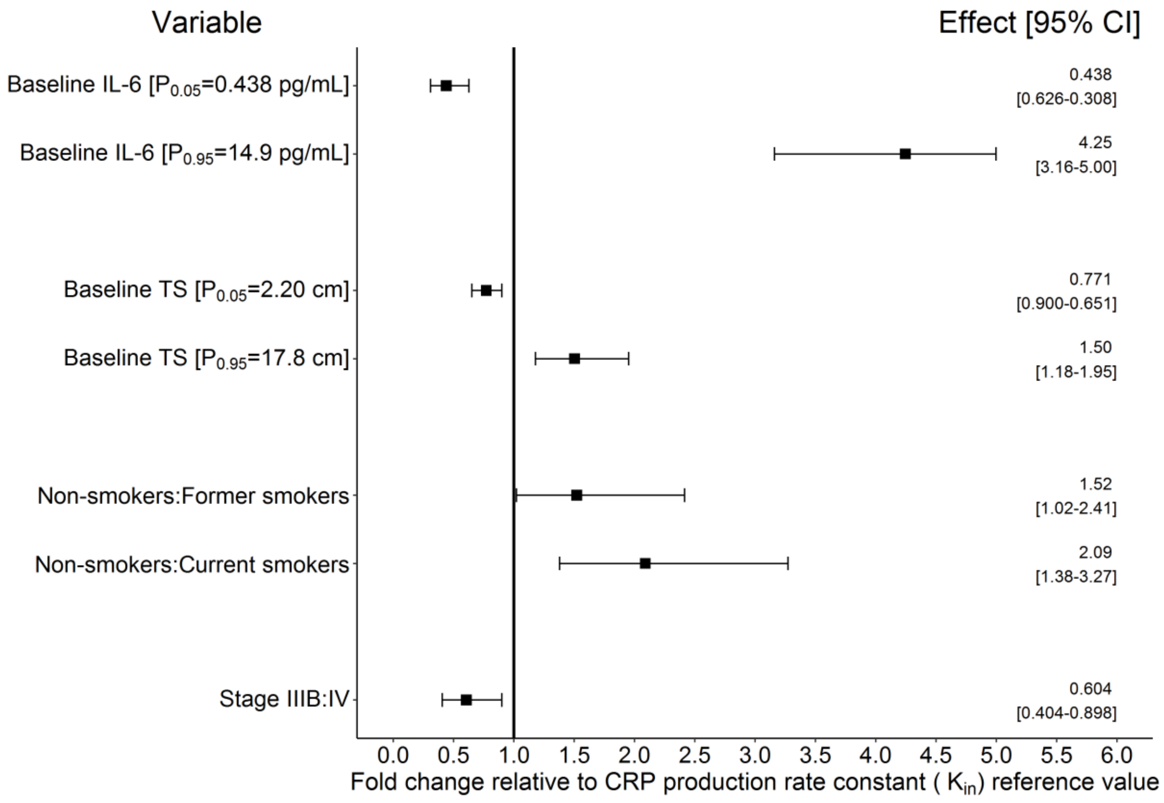

Figure S3. Forest plot of the impact of significant variables on C-reactive protein production rate constant (K_{in}) relative to the reference value 1 (bold vertical line, reference patient: non-smoker, disease stage IV, median baseline tumor size = 8.25 cm, median baseline IL-6 = 2.57 pg/mL). Effects of continuous variables (i.e., baseline IL-6, baseline tumor size) are shown at the 5th and 95th percentiles of the respective variable and effects of categorical variables (i.e., smoking status, disease stage) are shown relative to the reference category. Black boxes: variable effect; horizontal lines: 95% confidence intervals (CI); IL-6: interleukin-6; TS: tumor size.

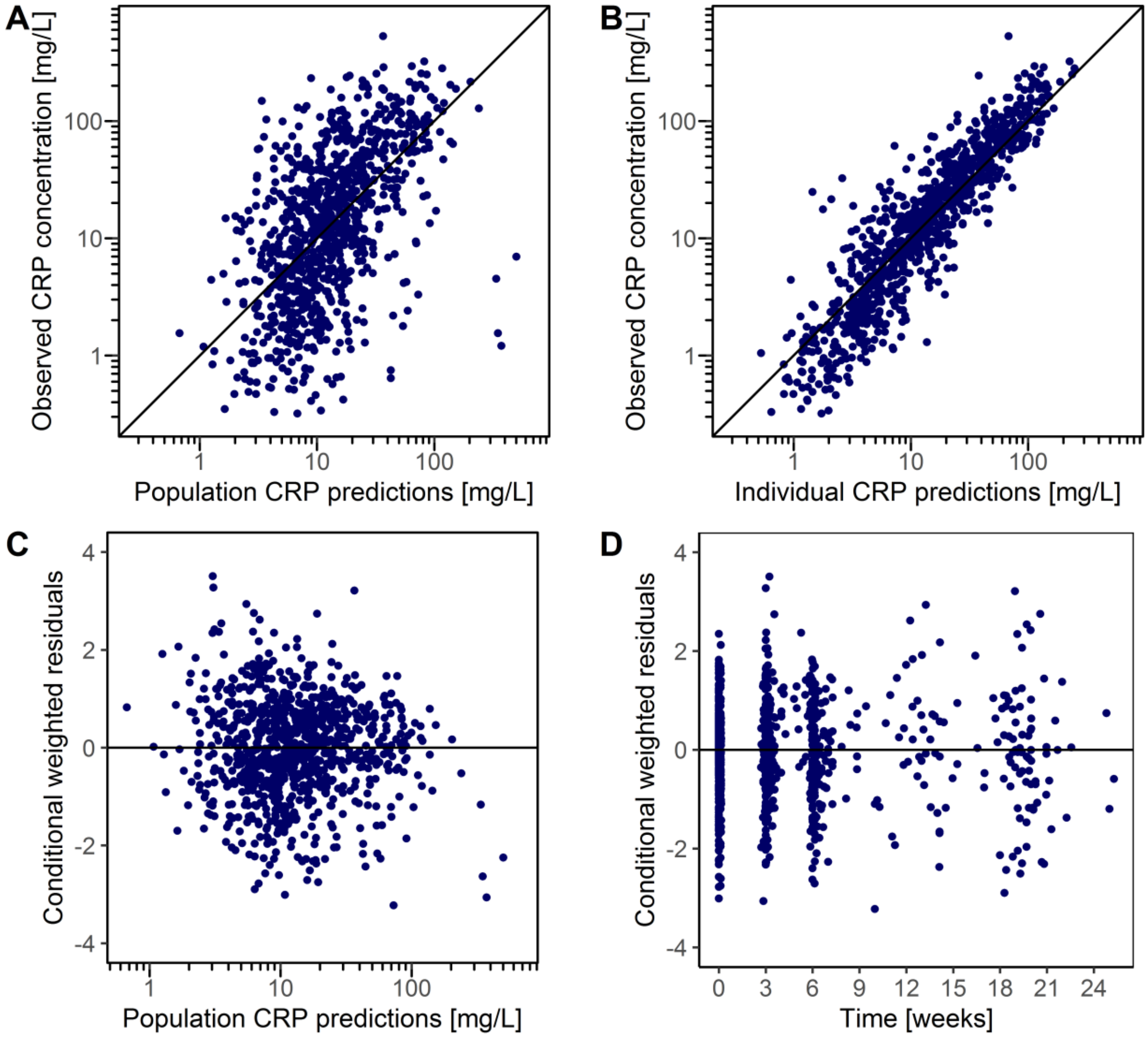

Figure S4. Basic goodness-of-fit plots of coupled tumor dynamics-CRP model **[A]** observations versus population predictions; **[B]** observations versus individual predictions; **[C]** conditional weighted residual versus population predications; **[D]** conditional weighted residuals versus time. Blue dots: data points; solid black line: line of identity of slope 1 or zero. CRP: C-reactive protein.

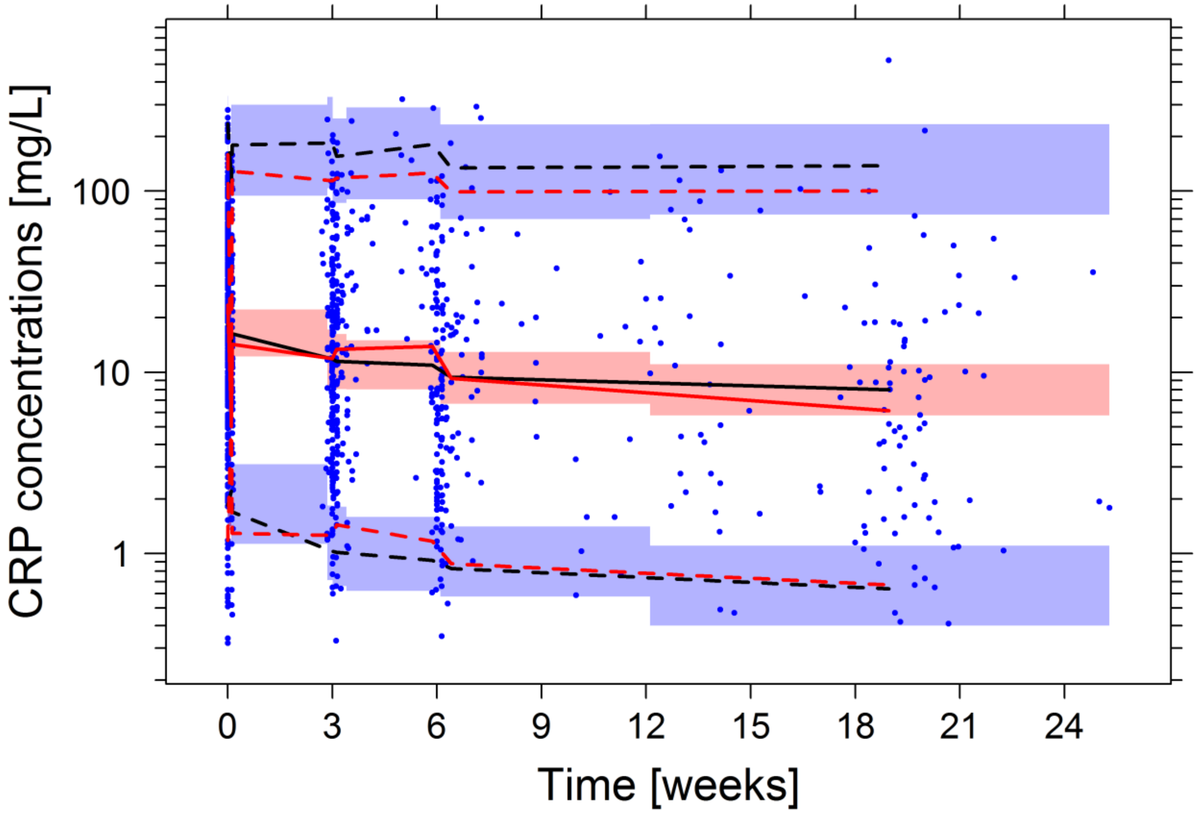

Figure S5. Visual predictive check (n = 250 simulations) of the coupled tumor dynamics-CRP model. Dots: observed CRP concentrations; solid red and black lines: median of the observed and simulated CRP concentrations, respectively; upper and lower dotted red and black lines: 5th and 95th percentiles of the observed and simulated CRP concentrations, respectively. Shaded areas: 95% confidence interval of the simulated percentiles. The number of bins was set to 8 with equal number of observations. CRP: C-reactive protein.

References

1. Lacroix BD, Friberg LE, Karlsson MO. Evaluation of IPPSE, an alternative method for sequential population PKPD analysis. J Pharmacokinet Pharmacodyn. 2012;39:177–93.

2. Rubin DB. Multiple imputation for nonresponse in surveys. John Wiley & Sons; 1987.

3. Dayneka NL, Garg V, Jusko WJ. Comparison of four basic models of indirect pharmacodynamic responses. J Pharmacokinet Biopharm. 1993;21:457–78.

4. Upton RN, Mould DR. Basic concepts in population modeling, simulation, and model-based drug development: Part 3-introduction to pharmacodynamic modeling methods. CPT Pharmacometrics Syst Pharmacol. 2014;3:1–16.

5. Vigushin DM, Pepys MB, Hawkins PN. Metabolic and scintigraphic studies of radioiodinated human C-reactive protein in health and disease. J Clin Invest. 1993;91:1351–7.

6. Jonsson EN, Karlsson MO. Automated covariate model building within NONMEM. Pharm Res. 1998;15:1463–8.

7. Lindbom L, Ribbing J, Jonsson EN. Perl-speaks-NONMEM (PsN) - A Perl module for NONMEM related programming. Comput Methods Programs Biomed. 2004;75:85–94.

8. Ojara FW, Henrich A, Frances N, Nassar YM, Huisinga W, Hartung N, et al. A prognostic baseline blood biomarker and tumor growth kinetics integrated model in paclitaxel/platinum treated advanced non-small cell lung cancer patients. CPT Pharmacometrics Syst Pharmacol [Internet]. John Wiley & Sons, Ltd; 2023;n/a. Available from: https://doi.org/10.1002/psp4.12937
